# Echinoids from the Tesero Member (Werfen Formation) of the Dolomites (Italy): implications for extinction and survival of echinoids in the aftermath of the end-Permian mass extinction

**DOI:** 10.7717/peerj.7361

**Published:** 2019-08-30

**Authors:** Jeffrey R. Thompson, Renato Posenato, David J. Bottjer, Elizabeth Petsios

**Affiliations:** 1 Department of Genetics, Evolution and Environment, University College London, University of London, London, United Kingdom; 2 Department of Earth Sciences, University of Southern California, Los Angeles, CA, United States of America; 3 Department of Geosciences, Baylor University, Waco, TX, United States of America; 4 Dipartimento di Fisica e Scienze della Terra, Università di Ferrara, Ferrara, Italy

**Keywords:** Sea urchins, Recovery, Bottleneck, Biodiversity, Triassic, Cidaroid

## Abstract

The end-Permian mass extinction (∼252 Ma) was responsible for high rates of extinction and evolutionary bottlenecks in a number of animal groups. Echinoids, or sea urchins, were no exception, and the Permian to Triassic represents one of the most significant intervals of time in their macroevolutionary history. The extinction event was responsible for significant turnover, with the Permian–Triassic representing the transition from stem group echinoid-dominated faunas in the Palaeozoic to Mesozoic faunas dominated by crown group echinoids. This turnover is well-known, however, the environmental and taxonomic distribution of echinoids during the latest Permian and Early Triassic is not. Here we report on an echinoid fauna from the Tesero Member, Werfen Formation (latest Permian to Early Triassic) of the Dolomites (northern Italy). The fauna is largely known from disarticulated ossicles, but consists of both stem group taxa, and a new species of crown group echinoid, *Eotiaris teseroensis* n. sp. That these stem group echinoids were present in the Tesero Member indicates that stem group echinoids did not go extinct in the Dolomites coincident with the onset of extinction, further supporting other recent work indicating that stem group echinoids survived the end-Permian extinction. Furthermore, the presence of *Eotiaris* across a number of differing palaeoenvironments in the Early Triassic may have had implications for the survival of cidaroid echinoids during the extinction event.

## Introduction

The end-Permian extinction saw the demise of several evolutionary lineages, and ushered in the taxonomic and ecological restructuring of marine invertebrate ecosystems that gave rise to the Modern Fauna (Sepkoski, 1984). The combined effects of thermal stress and hypoxia (Sun et al., 2012; Song et al., 2014; Penn et al., 2018; Petsios et al., 2019), ultimately precipitated by the emplacement of the Siberian Traps Large Igneous Province (Reichow et al., 2009; Burgess, Bowring & Shen, 2014), is hypothesized to have led to 96% species loss across faunas with differing palaeophysiology and palaeoecology (Raup, 1979; Bambach, 2006). The severity of the taxonomic loss led to bottlenecks and eventual evolutionary turnover across several groups, including ammonoid cephalopods (McGowan, 2004; Brayard et al., 2009; Pietsch et al., 2019), conodonts (Orchard, 2007), ostracod crustaceans (Crasquin & Forel, 2014), and the focus of this current study, the echinoids or sea urchins.

The end-Permian mass extinction represents one of the most significant intervals of time for understanding the macroevolutionary history of echinoids (Erwin, 1993; Erwin, 1994; Benton, 2003; Thompson et al., 2018). During the Palaeozoic, echinoid faunas were comprised predominantly of imbricate plated stem-group echinoids, with abundant columns of interambulacral and ambulacral plates. Almost all of the post-Palaeozoic echinoids, however, have a body plan constructed of only two columns of interambulacral and ambulacral plates (Erwin, 1993; Erwin, 1994; Kroh & Smith, 2010), indicating significant turnover during the Permian–Triassic interval. In the late Palaeozoic, echinoid faunas were comprised of the stem group families Archaeocidaridae, Lepidesthidae, Proterocidaridae, Cravenechinidae and Lepidocentridae (Jackson, 1912; Kier, 1958b; Kier, 1965; König, 1982; Thompson, Petsios & Bottjer, 2017) and the early crown group echinoids of the family Miocidaridae (Smith & Hollingworth, 1990; Thompson et al., 2015). By the early Mesozoic, only the miocidarids and proterocidarids remained (Hagdorn, 2018; Thuy, Hagdorn & Gale, 2017; Thompson et al., 2018; Pietsch et al., 2019). Indeed, only one genus, the miocidarid *Eotiaris*, is known from fossil representatives on both sides of the mass extinction event (Smith & Hollingworth, 1990; Thompson et al., 2015; Godbold et al., 2017), though phylogenetic analyses indicate more boundary crossers likely existed as ghost lineages (Smith, 2007; Thompson et al., 2018; Pietsch et al., 2019).

Though there is little doubt that the Permian–Triassic interval had a profound impact on echinoid macroevolution, the precise details surrounding extinction and duration of echinoid groups, especially on the genus and family levels, remain obscured. Here we describe in detail an echinoid fauna from the Tesero Member, Werfen Formation of Italy, including a new species of the genus *Eotiaris, Eotiaris teseroensis* n. sp., which has been mentioned several times in the literature (Broglio Loriga, Neri & Posenato, 1986; Broglio Loriga et al., 1988; Broglio Loriga et al., 1990; Posenato, 1988) but which has until now remained undescribed. In the Tesero section, the Tesero Member spans the Permian–Triassic Boundary (PTB) and contains a rich “mixed fauna” (Neri & Pasini, 1985; Broglio Loriga et al., 1988) which occurs within the end-Permian extinction interval (about three m thick) and above the extinction peak occurring in the lowermost Tesero Member (e.g., Chen et al., 2006; Groves et al., 2007; Posenato, 2009; Posenato, 2010; Clapham et al., 2013). In addition to the specimen of *Eotiaris*, we also describe additional disarticulated material from the Tesero Member which is attributed to both stem group and crown group echinoids. Analysis of the stratigraphic setting of these echinoid occurrences relative to the onset of the extinction event, and comparison of this fauna to other pre- and post-extinction echinoid faunas informs the nature and timing of faunal turnover in echinoid communities, and the palaeoenvironmental distribution of echinoids during the Permian–Triassic interval. This provides clues as to the nature and timing of echinoid extinction and survival during the end-Permian mass extinction (EPME).

**Figure 1:**
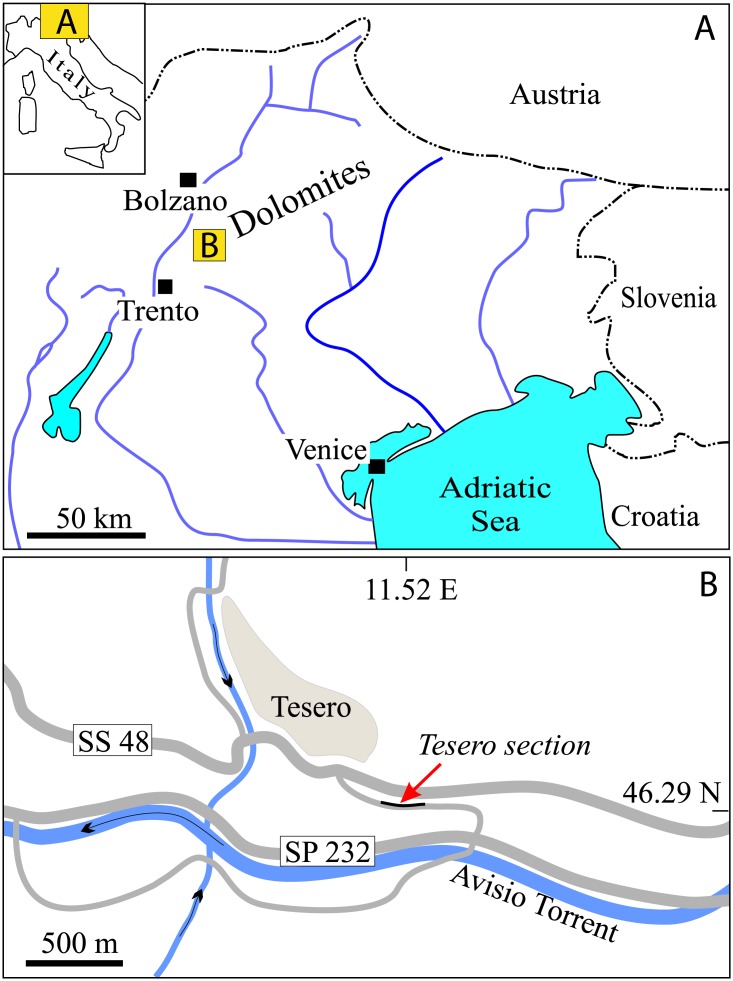
Geographic position of the Tesero section within (A) Italy and within (B) the Fiemme Valley, Trento Province.

### Geological and stratigraphical setting

Since the 19th century, the Dolomites (northern Italy; Fig. 1) have represented a reference area to study the EPME of the shallow marine biota. The Upper Permian is represented by the Bellerophon Formation, a thick sulphate-carbonate succession, which records marginal (sabkha) to shallow marine conditions (e.g., Massari & Neri, 1997). The upper part of the formation is characterised by black bioclastic limestone, which contains rich benthic assemblages dominated by calcareous algae, foraminifers, molluscs and brachiopods. The Bellerophon Formation is overlain by the Werfen Formation, which begins with the Tesero Member, a transgressive carbonate unit mostly composed of oolitic grainstones, mudstone and microbial limestone (e.g., Bosellini, 1964; Noé, 1987; Beretta et al., 1999; Farabegoli, Perri & Posenato, 2007; Groves et al., 2007; Jia et al., 2012). The basal centimetres of the Tesero Member record the peak of the EPME, while the PTB has been recognized a few decimetres above (e.g., 1.3 m above the formational boundary in the Bulla section; for comprehensive descriptions of the PTB succession of the Dolomites see Farabegoli, Perri & Posenato (2007), Posenato (2009) and Posenato (2010)).

**Figure 2:**
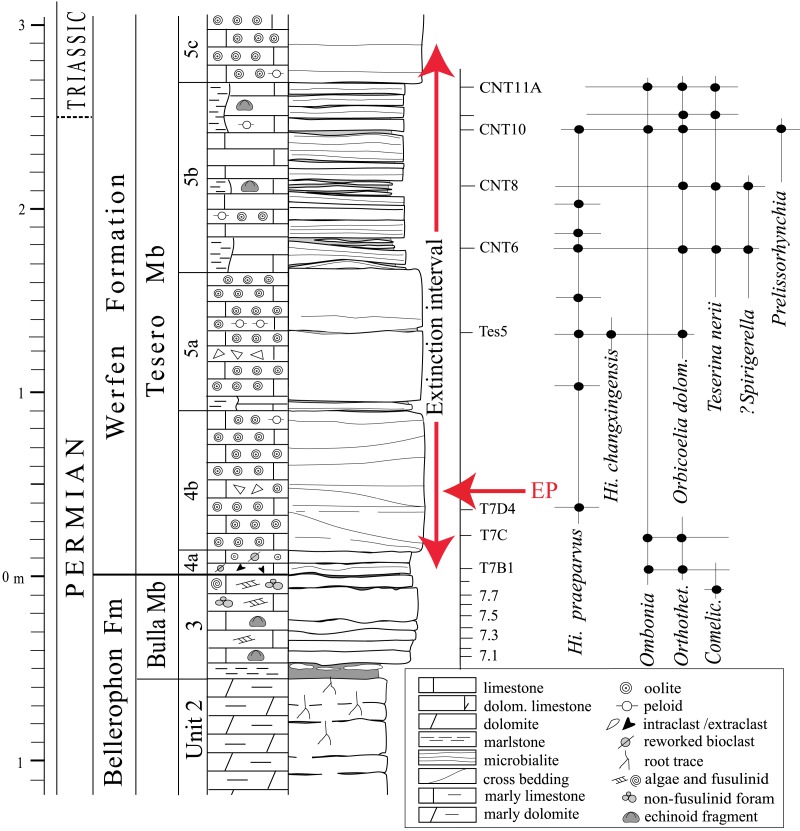
Stratigraphic column of the Tesero succession containing the formational and (supposed) erathem boundaries and the stratigraphic ranges of conodonts and brachiopods. The duration of the extinction interval is from Posenato (2010); the extinction peak (EP) refers to the extinction of the lagenide foraminifers at a confidence interval of 96% (from Groves et al., 2007). The echinoids studied herein were collected from beds CNT6 to CNT11A. Abbreviations: *Orthothet., Orthothetina*; *Comelic., Comelicania*; *Hi., Hindeodus* (modified from Posenato, 2009).

Conodonts are rare in the Tesero section (e.g., Farabegoli, Perri & Posenato, 2007). *Hindeodus changxingensis*, the marker of the last Permian biozone and proxy of the PTB (Metcalfe, Nicoll & Wardlaw, 2007), occurs 1.3 m above the Bellerophon–Werfen formational boundary (BWFB), while *Hindeodus parvus*, the marker of the PTB, appears here only at 11 m above the BWFB, after a 9 m interval lacking conodonts. For this reason, the *H. parvus* first occurrence in the Tesero section has been considered diachronous and younger than that of the Bulla section, another key section for studies of the PTB in the Dolomites. At Bulla, it appears at 1.3 m above the BWFB (Perri & Farabegoli, 2003). Following Posenato (2009) and Farabegoli & Perri (2012), the PTB in the Tesero section is tentatively placed in the upper part of unit 5b, slightly predating bed CNT11A (Fig. 2), where the peak of the articulate brachiopod *Teserina nerii* occurs. Therefore, the echinoid bearing beds of the Tesero section probably have an age encompassing the PTB.

The Tesero section, easily accessible because of its location along the road connecting the village of Tesero with the main road along the bottom of the Avisio Valley (Fig. 1), is among the most studied PTB successions in the Dolomites. It was first described by Leonardi (1935), and carefully analysed over the course of the last few decades from palaeontological, sedimentological and geochemical points of view (e.g., Bosellini, 1964; Neri & Pasini, 1985; Noé, 1987; Broglio Loriga et al., 1988; Magaritz et al., 1988; Wignall & Hallam, 1992; Beretta et al., 1999; Perri & Farabegoli, 2003; Chen et al., 2006; Groves et al., 2007; Posenato, 2009; Spina et al., 2015; Foster et al., 2017). The section became famous globally after the discovery of a “mixed brachiopod and mollusc fauna” located about 1.5–2.8 m above the BWFB, formerly considered to be coincident with the PTB (Neri & Pasini, 1985).

At Tesero, the black bioclastic limestone of the Bellerophon Fm (Bulla Member) is only about 50 cm thick (Fig. 2, unit 3). It overlies light-grey, marly dolostone representing peritidal environments with root traces and mud-cracks, recording short-term subaerial exposure, in the underlying Bellerophon Formation unit (unit 2). The Tesero Member begins with a basal oolitic bedset, about 90 cm thick (units 4a, 4b), the middle of which records the extinction peak of lagenide foraminifers (Groves et al., 2007). It is followed by an interval of ca. 180 cm (units 5a, 5b) characterised by wide lateral facies variability, composed of oolitic and bioclastic grainstone/packstone, marly interlayers and lenses, mudstone and microbialite dm-size mounds (Beretta et al., 1999, fig. 4). The marlstone is dominated by the articulate brachiopods *Orbicoelia dolomitensis* Chen and *Teserina nerii* Posenato (Broglio Loriga et al., 1988; Chen et al., 2006; Posenato, 2009; Posenato, 2011), which are respectively abundant in the lower and upper “mixed fauna beds”. It is these same “mixed fauna beds” from which the echinoids described herein were collected.

In the upper Palaeozoic of northern Italy, two species of echinoid, *Archaeocidaris tirolensis* Stache, 1876 and *Archaeocidaris ladina* Stache, 1877 have been named from the Bellerophon Formation. These named taxa are, however, nomina nuda or indeterminate, respectively (Jackson, 1912) and are based off of disarticulated, fragmentary material. In the Tesero section, echinoid remains are abundant in the black limestone of the Bellerophon Fm (Bulla Mb), but cannot be extracted from the matrix because they are embedded in hard, cemented, limestone. The material studied herein has been collected from unit 5b of the Tesero Member (Fig. 2), where plates and spines are preserved on microbial bed surfaces or as isolated specimens extracted from washed residues obtained from disaggregation and sieving of the marlstones between the microbialites. Higher in the Werfen formation, echinoid spines are recorded within the Siusi Member (late Induan) (Broglio Loriga, Masetti & Neri, 1983, 1983, pl. 46).

## Materials & Methods

Terminology and classification for systematics follows Smith (1984), Kroh & Smith (2010), and Märkel (1979). Taxonomic methodology follows Lewis & Donovan (2007). Because most of the material dealt with herein is preserved as disarticulated ossicles, we chose to group taxa together in such a way as to minimize the number of discrete taxa. Thus all interambulacral plates and spine material with a prominent milled ring is grouped together with *Eotiaris teseroensis* n.sp., and all spines representing putative stem group echinoids are grouped together as indeterminate stem group echinoids. All disarticulated lantern elements and fragmentary spines lacking a proximal end, which cannot be attributed to either of the other taxonomic concepts used herein, are treated as indeterminate echinoids. All material is deposited in the “Museo di Paleontologia e Preistoria Piero Leonardi” of Ferrara University (MPL).

The electronic version of this article in Portable Document Format (PDF) will represent a published work according to the International Commission on Zoological Nomenclature (ICZN), and hence the new names contained in the electronic version are effectively published under that Code from the electronic edition alone. This published work and the nomenclatural acts it contains have been registered in ZooBank, the online registration system for the ICZN. The ZooBank LSIDs (Life Science Identifiers) can be resolved and the associated information viewed through any standard web browser by appending the LSID to the prefix http://zoobank.org/. The LSID for this publication is: urn:lsid:zoobank.org:pub:5B9E9AE3-B43E-44D0-99BE-F92C979CF325. The online version of this work is archived and available from the following digital repositories: PeerJ, PubMed Central and CLOCKSS.

### Systematic palaeontology

**Table:** 

Class Echinoidea Leske, 1778
Subclass Cidaroidea Smith, 1984
Family Miocidaridae Durham & Melville, 1957

**Type genus.** *Miocidaris* Döderlein, 1887

**Other genera.** *Eotiaris* Lambert, 1899*, Couvelardicidaris* Vadet, 1991, *Procidaris* Pomel, 1883

Genus *Eotiaris* Lambert, 1899

**Type species.** *Cidaris keyserlingi* Geinitz, 1848, from the Wuchiapingian Zechstein of Germany and the United Kingdom.

**Other species.** *Eotiaris guadalupensis* Thompson 2017 in Thompson, Petsios & Bottjer, 2017 from the Roadian and Capitanian of west Texas and *E. connorsi* Kier, 1965 from the Capitanian of West Texas.

**Diagnosis.** Miocidarid with small test. Interambulacral plates imbricate adapically. Areoles confluent at or below ambitus.

**Figure 3:**
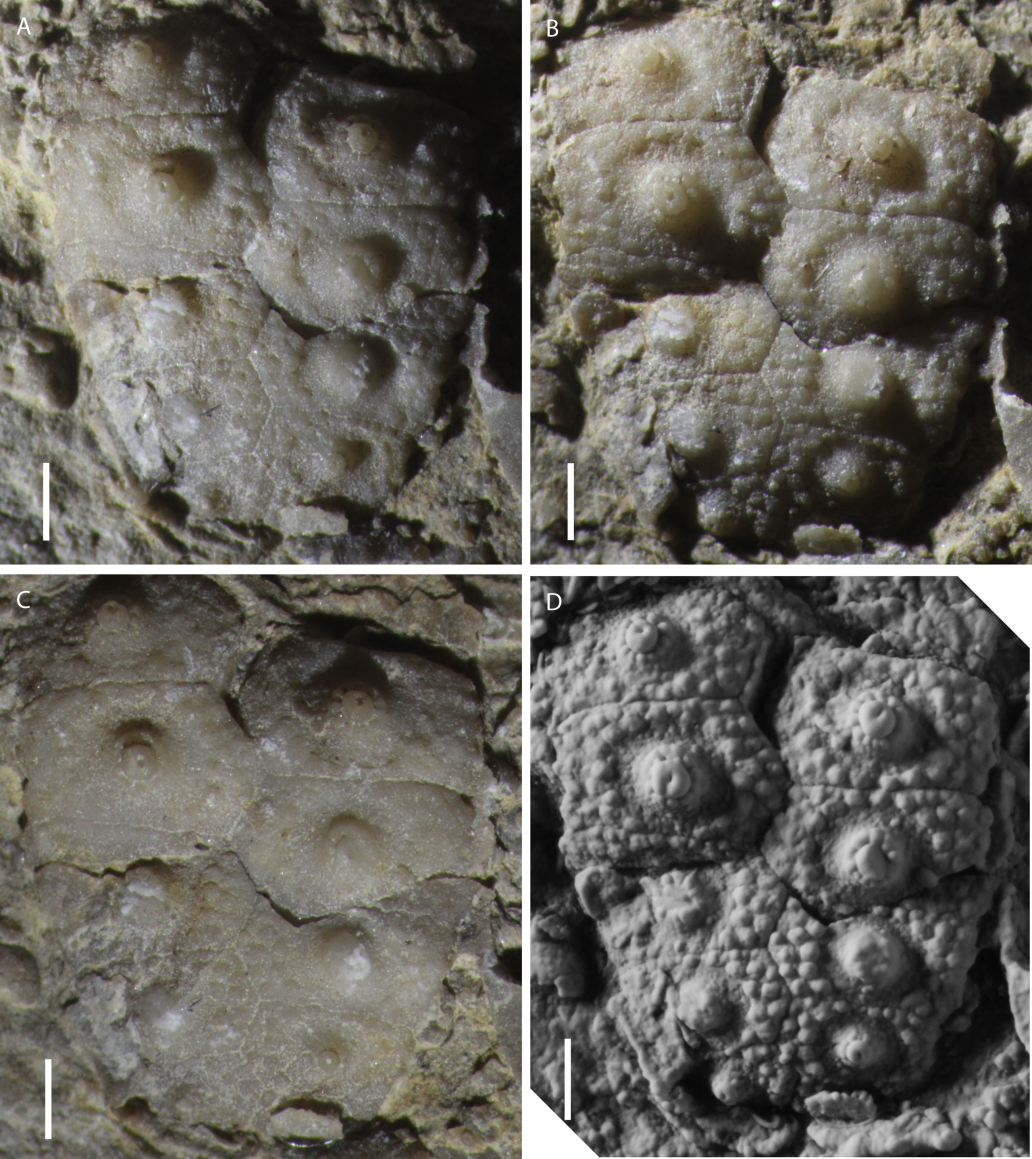
Photos of specimen MPL 8651-1 of *Eotiaris teseroensis.* Photos of specimen MPL 8651-1 of *Eotiaris teseroensis*. (A-D) show the same specimen, with different angles and amounts of lighting. Note the crenulate tubercles, and confluence of areoles. The specimen in (D) has been coated with ammonium chloride prior to photography. Photo credit for A-C to Jeffrey Thompson and D to Renato Posenato.

**Occurrence.** Wuchiapingian of Germany, the U.K. and Roadian and Guadalupian of Texas.

**Table:** 

**Figure 4:**
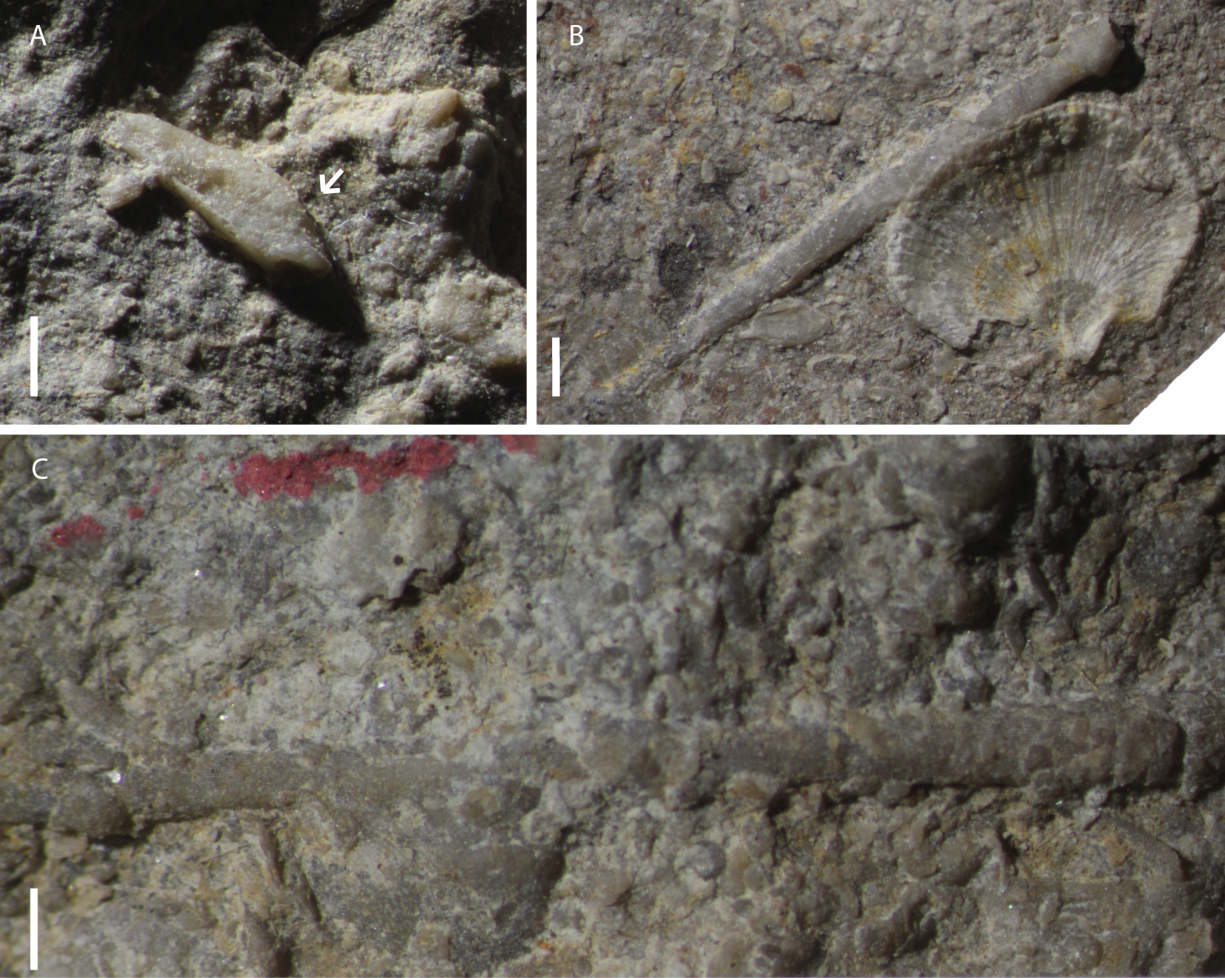
Plates and lantern elements attributed to *Eotiaris teseroensis* or indeterminate. (A) Specimen MPL 8651-2, a disarticulated hemipyramid. Note the shallow foramen magnum indicated with arrow. (B) Specimen MPL 8652, disarticulated spine from *E. teseroensis*. (C) Specimen MPL 8659, disarticulated spine from *E. teseroensis.* Photo credit to Jeffrey Thompson.

**Figure 5:**
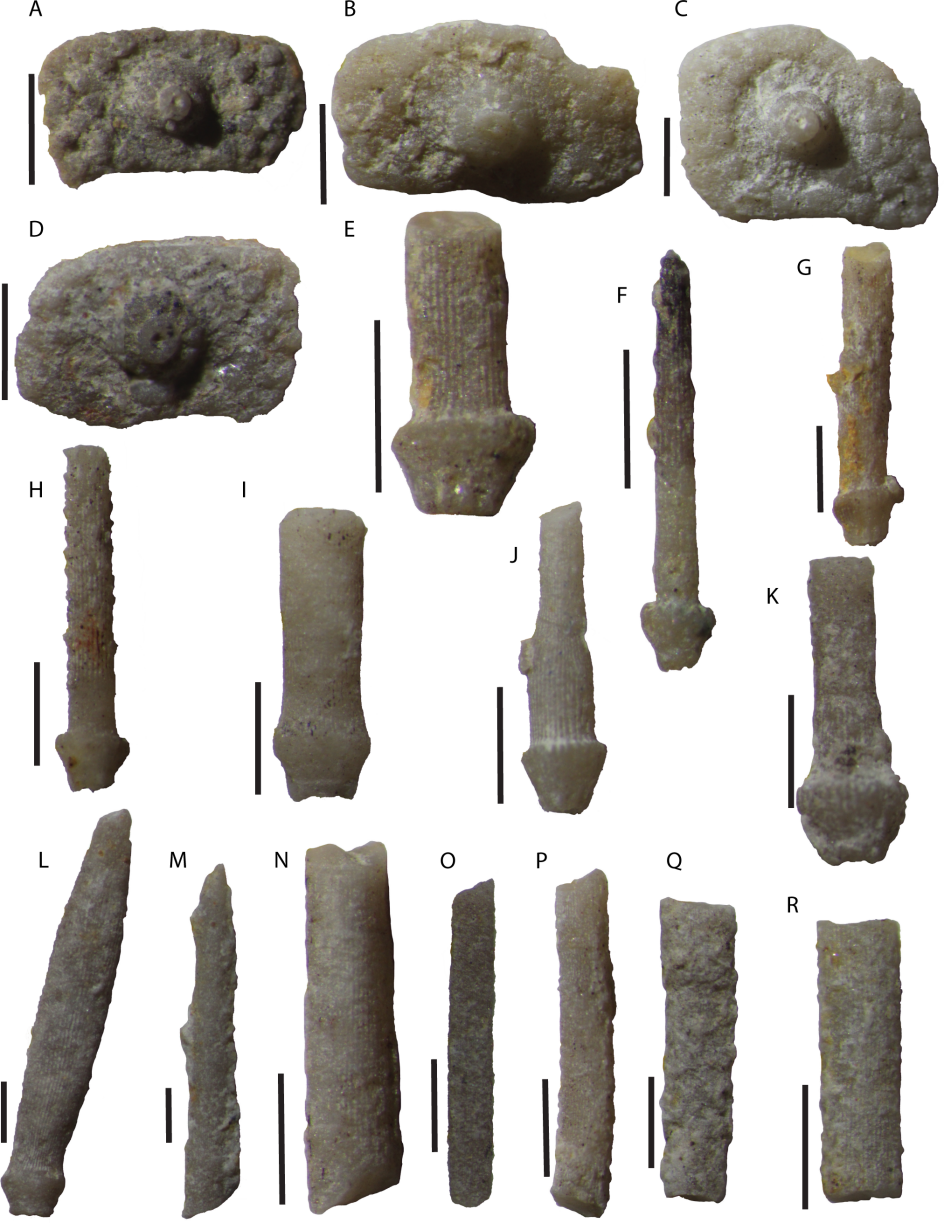
Disarticulated echinoid interambulacral plates and spines from the Tesero Member of the Werfen Formation. Interambulacral plates (A–D) and spines with distinct milled ring (E–L) are attributed to *Eotiaris teseroensis*, while spine fragments (M–R) are indeterminate. (A) Specimen MPL 8653-1, disarticulated interambulacral plate. (B) Specimen MPL 8656-2, fragmentary interambulacral plate from sample. (C) Specimen MPL 8656-1, fragmentary interambulacral plate; note non-confluent areole. (D) Specimen MPL 8657-5, interambulacral plate; note crenulate tubercle. (E) specimen MPL 8654-2, fragmentary spine; note prominent milled ring. (F) Specimen MPL 8655-1, spine; small spinules on distal tip of spine. (G) Specimen MPL 8653-5, fragmentary spine. (H) Specimen MPL 8653-3, incomplete spine; note small spinules along spine shaft. (I) Specimen MPL 8657-4, spine base and proximal shaft. (J) Specimen MPL 8657-3, spine. (K) Specimen MPL 8658-5, spine base and proximal spine. (L) Specimen MPL 8657-6, spine; note slightly fusiform morphology of spine. (M) Specimen MPL 8657-2, spine fragment. (N) Specimen MPL 8654-3, spine fragment. (O) Specimen MPL 8658-4, spine fragment. (P) Specimen MPL 8653-4, spine fragment. (Q) Specimen MPL 8658-2, spine fragment. (R) Specimen MPL 8658-7, spine fragment. All scale bars one mm. Photo credit to Jeffrey Thompson.

*Eotiaris teseroensis* n sp.
Figs. 3, 4B, 4C, 5A–5L
urn:lsid:zoobank.org:act:C14A9C9C-90FB-4F41-9A7B-AF5D0C43A326
1986 ?*Miocidaris* sp. Broglio Loriga et al. p. 9; pl. 1, fig. 3.
1988 ?*Miocidaris* sp. Broglio Loriga et al. p. 26; pl. 2 fig. 10.
1988 *Miocidaris* sp. Posenato p. 40; pl. 2, fig. 8.
1990 *Miocidaris* sp. Broglio Loriga et al. pl. 1, fig. 5.

**Diagnosis.** *Eotiaris* with only first one or two most adoral areoles confluent. Spines long, thin, straight and cylindrical. Some spines striate and some with small distally facing spinules.

**Etymology.** After the locality Tesero, where the holotype was collected.

**Holotype.** The holotype is specimen MPL 8651-1 (Figs. 3A–3D).

**Material.** In addition to the holotype, a number of disarticulated spines and interambulacral plates are attributed to this taxon. These are MPL 8652, MPL 8659, MPL 8653-1, MPL 8656-2, MPL 8656-1, MPL 8657-5, MPL 8655-1, MPL 8653-5, MPL 8653-3, MPL 8657-4, MPL 8657-3, MPL 8658-5, MPL 8654-2, and MPL 8657-6.

**Occurrence.** This species is known from the latest Changhsingian –early Induan Tesero Member at the Tesero section, Dolomites of Italy.

**Description.** Test regular and small. Only a single interambulacral area 6.1 mm in width is known from specimen MPL 8651-1 (Fig. 3), but using the calculation of Smith & Hollingworth (1990), the test diameter would likely have been about 13.5 mm. Hemipyramids and rotulae that may belong to this taxon are described below as Echinoidea, gen. et sp. indet. The peristomial plating, apical plating and details of the perignathic girdle are unknown.

The interambulacrum is composed of two columns of plates (Fig. 3). At and below the ambitus the plating appears to be rigid, while plates above the ambitus are missing. Five plates are preserved in each column, though more were undoubtedly present. These missing adapical plates may have been imbricate as is the case for other species in the genus (e.g., *E. keyserlingi*). Interambulacral plates are pentagonal, ranging from 1.5 to 2.1 times wider than high (Table 1). Interambulacral tubercles are perforate and crenulate, slightly sunken. The one or two most adoral plates with confluent areoles adorally and adapically (Fig. 3). One disarticulated plate attributed to this taxon without confluent areoles (Fig. 5C) so more adapical plates were likely without confluent areoles. Areoles at the ambitus are circular, from 1.4 to 1.7 mm high and 1.5 to 1.8 mm wide (Table 1). Each plate is covered with numerous imperforate, non-crenulate secondary tubercles, which are arranged in a closest-packing arrangement and cover the entire extrascrobicular area. There are about 40 secondary tubercles on the largest ambital plates, and about 25 on the second most adoral plate (Fig. 3). At the ambitus there are three rows of scrobicular tubercles between the areoles and the adambulacral suture and three rows between the areole and interradial suture. On the second most adoral plates, there are about two rows of scrobicular tubercles between the areole and adambulacral suture and one or two between the areole and interradial suture. Scrobicular tubercles differentiated into slightly distinct scrobicular ring (Figs. 5A, 5C).

**Table 1:** Measurements from select interambulacral plates of *Eotiais teseroensis* from specimen MPL 8651-1.

**Plate**	**1**	**2**	**3**	**Mean**
**Plate height (mm)**	1.5	2.1	1.7	1.8
**Plate width (mm)**	3.2	3.2	2.8	3.1
**Plate width/height**	2.1	1.5	1.6	1.8
**Areole height (mm)**	1.5	1.7	1.4	1.5
**Areole width (mm)**	1.8	1.7	1.5	1.7
**Areole width/height**	1.2	1.0	1.1	1.1

Spines attributed to this species are long, thin. They are straight and cylindrical or slightly fusiform. Some spines striate and lacking ornamentation (Figs. 5J, 5L) and some with small distally facing spinules located distally (Figs. 5F–5H). All spines with a well-defined milled ring (Figs. 5E–5L). Straight spines widest at the milled ring, fusiform spines widest at about midway their length (Fig. 5L). Longest spine fragment is 17.4 mm in length, though this is not associated with any test thus the spine length relative to test size is unknown. Most other complete spines are much shorter.

**Remarks.** The two columned interambulacral area, morphology of spines, confluence of areoles, imbricate adradial suture and imbricate adapical interambulacral plating identify this species as a member of the genus *Eotiaris*. The specimens described herein are morphologically similar to many specimens of *Eotiaris keyserlingi* (Geinitz, 1848) known from the Wuchiapingian Zechstein of Germany (Reich, 2007), the United Kingdom (Smith & Hollingworth, 1990) and reported from Poland (Peryt et al., 2016). *E. teseroensis* differs from *E. keyserlingi* with respect to the confluence of it’s areoles. While only the first one or two areoles of *E. teseroensis* are confluent, in *E. keyserlingi*, about the four most adoral interambulacral plates bear confluent areoles. Other than the features of the interambulacral plates, the main differentiating feature of species of *Eotiaris* is the morphology of the spines. Both *E. keyserlingi* from the Zechstein and *E. teseroensis* described herein have spines which are smooth, taper distally and bear small spinules at their distal end (Compare Figs. 5F, 5H with fig. 4.6 in Smith & Hollingworth, 1990). There apparently exists much variability in the spines attributed to *E. keyserlingi*, as those shown in Fig. 3C by Reich (2007) bear many more spinules along their proximal shaft than those in fig. 4.6 of Smith & Hollingworth (1990). Furthermore, some spines attributed to *E. keyserlingi* appear to show no spinules, and are instead smooth for their entire length. Many echinoids, both extant and fossil, show aboral to oral variability in spine morphology along their test (Mortensen, 1928; Schneider, Sprinkle & Ryder, 2005), thus it should be no surprise that this is found in *E. keyserlingi*. In our washed samples and on bedding planes, we also find variability in spine morphology, from thin striate spines with distal spinules to fusiform spines lacking ornamentation (Figs. 5F–5L). *E. teseroensis* differs substantially from *E. guadalupensis* Thompson in Thompson, Petsios & Bottjer, 2017 from the Roadian to Capitanian of west Texas in the morphology of its spines. *E. guadalupensis* has spines covered with numerous spinules which are straight to fusiform to clavate or bulbous in shape (Thompson et al., 2015; Thompson, Petsios & Bottjer, 2017). The spines attributed to *E. teseroensis,* however, are much less wide and do not have the large, bulbous or clavate morphology or distinct spinules of *E. guadalupensis. E. teseroensis*, *E. keyserlingi,* and *E. guadalupensis* all differ from *E. connorsi* Kier, 1965 in having narrower interambulacral plates, denser secondary tuberculation on their interambulacral plates, and more rigidly sutured plating adorally. Phylogenetic analyses also indicate that *E. connorsi* may be more basal than the other species of *Eotiaris* (Thompson et al., in press). Despite the fact that the echinoids described herein largely differ morphologically from *E. keyserlingi* only with respect to the number of confluent areoles adorally, there also exist biogeographic differences between the Zechstein basin and the tropical Tethyan carbonates of northern Italy (Mei & Henderson, 2001).

**Table:** 

**Figure 6:**
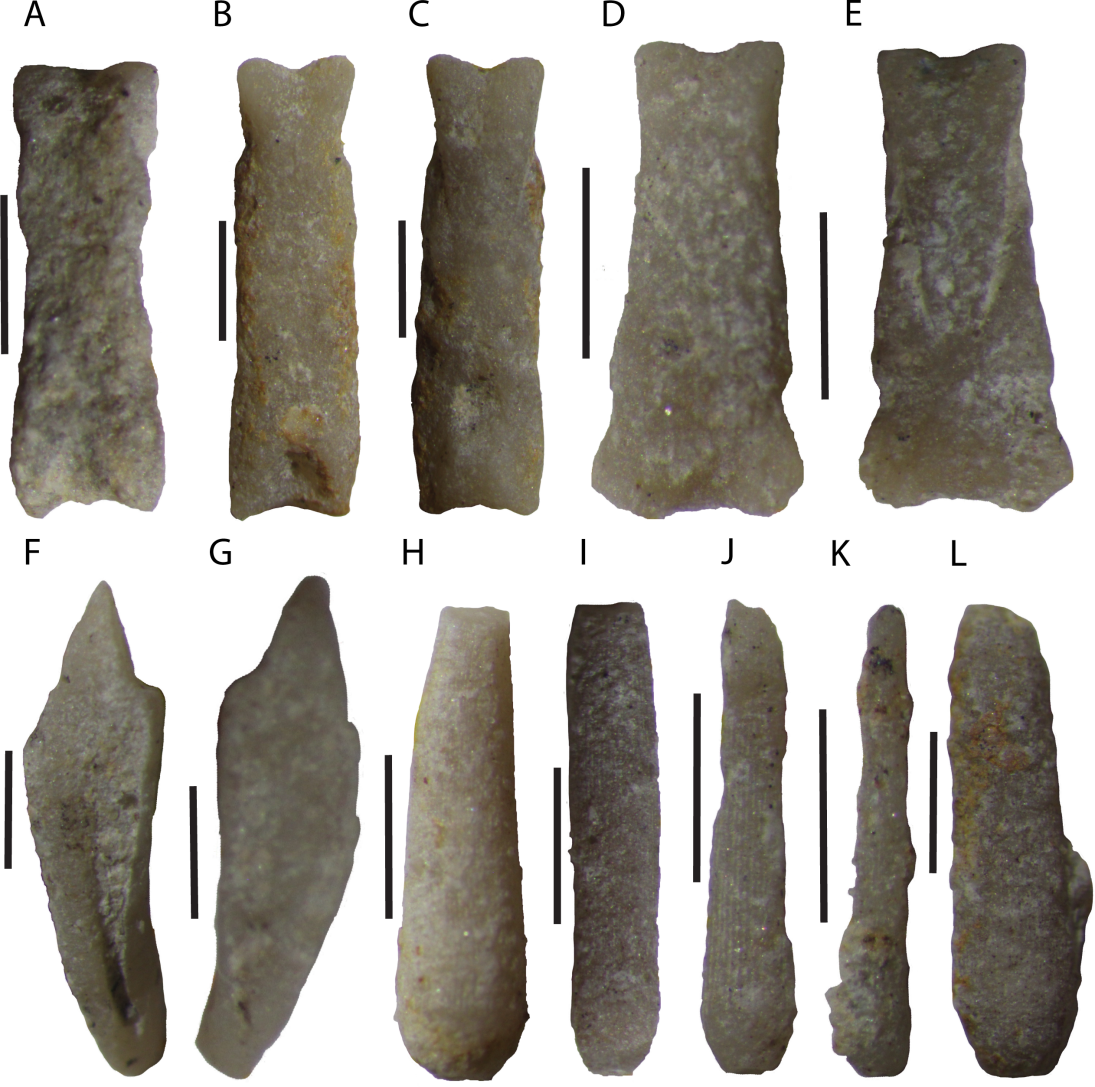
Disarticulated echinoid Aristotle’s lantern elements and spines from the Tesero Member of the Werfen Formation. Lantern elements are indeterminate (A–G), while spines are attributed to stem group echinoids (H–L). (A) Specimen MPL 8654-1, disarticulated rotula from sample TCn 10. (B) Specimen MPL 8657-8, disarticulated rotula from sample CNT11A . (C) Same as B. (D) Specimen MPL 8658-1, disarticulated rotula from sample CNT6. (E) Same as D. (F) Specimen MPL 8658-8 disarticulated hemipyramid from sample CNT6. (G) Specimen MPL 8657-1, disarticulated hemipyramid from sample CN T11A. (H) Specimen MPL 8653-2, disarticulated spine. (I) Specimen 8657-7. (J) Specimen MPL 8658-6, disarticulated spine from sample CNT6. (K) Specimen MPL 8655-2, disarticulated spine. (L) Specimen MPL 8658-3, disarticulated spine from sample CNT6. In spines attributed to stem group echinoids (H-L) note lack of milled ring and rounded, smooth acetabulum. All scale bars 1 mm. Photo credit to Jeffrey Thompson.

Stem Group Echinoidea, gen. et sp. indet.
Figs. 6H–6I

**Material.** Five disarticulated spines: MPL 8653-2, 8657-7, MPL 8658-6, MPL 8655-2, and MPL 8658-3.

**Description.** This taxon is represented only from disarticulated spines (Figs. 6H–6I). These spines are striated and peg-like (up to 0.7 mm wide) and taper distally. They lack any visible ornamentation such as spinules and are widest where the spine meets the base. The acetabulum is round and perforated and they lack a milled ring (Figs. 6H–6I).

**Remarks.** Disarticulated spines recovered from washed residues display a morphology that is inconsistent with identification as crown group echinoids in the Palaeozoic or Early Mesozoic. These spines lack a milled ring (Figs. 6H–6I), a character which is found in many regular crown group echinoids, and appears to have first evolved in archaeocidarid echinoids during the Late Palaeozoic (Thompson et al., in press). Permian taxa with primary spines lacking a milled ring include *Pronechinus cretensis* König, 1982, *Pronechinus anatoliensis* Kier, 1965 both members of the Proterocidaridae, and *Meekechinus elegans* Jackson, 1912 of the Lepidesthidae. The stem group echinoid *Yunnanechinus loupingensis* Thompson et al., 2018 from the Anisian of Yunnan province, China and *Lazarechinus mirabeti* Hagdorn, 2018 from the Anisian of the French Muschelkalk (Thuy, Hagdorn & Gale, 2017) also display spines lacking a milled ring. All Permian crown group echinoids have spines with a milled ring (Kier, 1965; Smith & Hollingworth, 1990; Thompson et al., 2015; Thompson, Petsios & Bottjer, 2017) and this feature is also found in Permian archaeocidarids (Kittl, 1904; Boos, 1929; Kier, 1958b; Luepke, 1976; Simpson, 1976; Thompson, Petsios & Bottjer, 2017) and Lower and Middle Triassic cidaroids (Kier, 1968b; Kier, 1977; Hagdorn, 1995).

Because other ossicles representing stem group echinoid coronal plates were not recovered from washed residues, however, we note that it is necessary to treat the stem group nature of this material with caution.

**Table:** 

Echinoidea, gen. et sp. indet.
Figs. 4A, 5M–5R, 6A–6G

**Material.** Three disarticulated rotulae (MPL 8654-1, MPL 8657-8, and MPL 8658-1), and three disarticulated hemipyramids (MPL 8651-2, MPL 8658-8, and MPL 8657-1). Furthermore, we include numerous fragmentary spines (MPL 8657-2, MPL 8654-3, MPL 8658-4, MPL 8653-4, MPL 8658-2, MPL 8658-7) whose taxonomic affinities are indeterminate.

**Description.** This material consists of numerous disarticulated elements of the Aristotle’s lantern and fragmentary spines which are indeterminate. The lantern elements recovered include rotulae and hemipyramids (Figs. 4A, 6A–6G). Three rotulae were recovered, which are shown in Figs. 6A–6E. The rotulae are bilaterally symmetrical, dorso-ventrally flattened, roughly rectangular in shape, and ranging from about two to five mm in length. All rotulae collected are hinge type (Märkel, 1979). Two morphotypes are known, hereby referred to as morphotypes A (Figs. 6A–6C) and B (Figs. 6D–6E). In photographs, all rotulae are oriented with the adaxial end (the end closest to the center of the lantern) oriented towards the bottom of the page.

Mophotype A is about equally wide throughout its width, except for the abaxial end, which narrows slightly passing from the epicondyles to the condyles. The dorsal surface is smooth throughout most of its length, with the exception of the abaxial end which ends in two slight bulges, which are the condyles. At the adaxial end of the dorsal surface, there is a shallow depression (Figs. 6A, 6C) in which the adaxial end of the compass would have rested. On the ventral surface of the rotula, slight indentations representing fossae are present at the adaxial end. At the abaxial end, the narrowing the condyles passing adaxially is clear (Fig. 6B) The abraded nature of the material makes it difficult to determine the location of rotular muscle scars.

Morphotype B is much wider at its adaxial end than abaxial end (Figs. 6D–6E). The dorsal side is smooth, and the condyles not very distinct. At the adaxial end, there is a small indentation, where the compass would rest. Also at the adaxial end, there is a slight constriction. On the ventral side, the condyles and epicondyles are much clearer (Fig. 6E) and two lateral bulges toward the adaxial end are the middle and inner fossae. Between these fossae are small indentations, which probably represent the rotular muscle scars.

Hemipyramids are small, no more than four mm tall. The foramen magnum is shallow, no more than 1/5 the height of the pyramid (Figs. 6F, 6G). A thickened wing edge is present along the edge of the hemipyramids (Fig. 6F) though this is only obvious in specimen MPL 8658-8.

The spines included herein are finely striate or covered in small rough spinules (Figs. 5M–5R). They range in length from about two mm to longer than five mm in length. They are circular in cross section. The spines are straight or show slight curvature in their orientation and many appear as small cylinders (Figs. 5N, 5P, 5R).

**Remarks.** The two rotulae morphotypes described herein; morphotype A and morphotype B, may represent variation within a single species, or, more likely, may represent rotulae from two different taxa. The two morphotypes differ most obviously in their general shape, with the adaxial end of morphotype B being much larger than that of A (compare Figs. 6A–6C to Figs. 6D–6E). Additionally in morphotype A, it is difficult to distinguish the precise location and number of fossae, while in morphotype B, the two symmetrical bulges located towards the adaxial end of the ventral side likely represent the inner and middle fossae. Insomuch as both of these morphotypes are hinge-type, they are similar to other rotulae reported from the Palaeozoic and Triassic. The rotula figured by Lewis & Ensom (1982) from the Mississippian *Archaeocidaris whatleyensis* Lewis & Ensom, 1982 is similar in its general rectangular shape, and in having a hinge joint, to the rotulae described herein. Furthermore, the rotula of the Anisian–Ladinian *Triadotiaris grandaeva* (v. Alberti, 1834), is similar to our morphotype 2 in widening adaxially (Hagdorn, 1995, fig. 5).

Hoare & Sturgeon (1976) figured three different rotulae in their study of echinoid ossicles from the Pennsylvanian Vanport Limestone of Ohio. Our morphotype B is similar to their figures 1 and 2 from their plate 2, while our morphotype A is similar to their plate 2, figure 3, albeit less narrow. In their study they attributed their disarticulated ossicles to two taxa, an archaeocidarid and a lepidesthid. Though the family level differences in rotula morphology are not well-known in stem group echinoids, it is possible that our two morphotypes represent a stem group and early crown group echinoid. Determining if this is the case is further complicated, however, in that all stem group echinoids where the rotulae are well-known, including *Archaeocidaris* M’Coy, 1844, *Nortonechinus* Thomas, 1920, and *Polytaxicidaris* Kier, 1958a and early crown group echinoids, such as *T. grandaeva* have hinge-type lanterns (Thomas, 1921; Kier, 1965; Märkel, 1979; Lewis & Ensom, 1982; Hagdorn, 1995). Further sampling of specimens where detailed morphology of rotulae, and of plates that are taxonomically informative at the family level (Thompson & Denayer, 2017), will be necessary to tie the details of differences in rotulae morphology to higher-taxonomic level classification in stem group echinoids.

The hemipyramids described herein are similar to those drawn by Smith & Hollingworth (1990) for *E. keyserlingi*. Both lanterns have shallow foramen magna, as is seen in crown group cidaroids. They are also much smaller than other hemipyramids potentially belonging to cidaroids described from the Capitanian of Texas (Thompson, Petsios & Bottjer, 2017). While the hemipyramids described by Thompson, Petsios & Bottjer (2017) were much too large to be attributed to the co-occurring cidaroid *E. guadalupensis*, the hemipyramids described herein are not much taller than a few millimeters, and could well belong to *E. teseroensis.* Though this may be the case, the incomplete nature of these hemipyramids and the fact that stem group echinoids likely also occur from the Tesero member precludes definitive attribution of these hemipyramids to a particular taxon.

The spines included herein are indeterminate given the state of their preservation, and could belong to any of the discussed taxa herein. Given the length and cylindrical nature of these spines, they may belong to *Eotiaris teseroensis*. Given their incomplete nature, however, we refrain from assigning them formally to that taxon.

## Discussion

### Palaeoecological setting of the Tesero echinoids

The echinoid fauna reported herein is remarkable in that it occurs within the end-Permian extinction interval, about 2 m above the horizon representing the onset and peak of the EPME and about 0.4 m below the disappearance of the last stenotopic marine taxa (e.g., calcareous algae; e.g., Groves et al., 2007; Posenato, 2010; Fig. 2). It thus provides a glimpse into echinoid faunas and their palaeoecological setting in the immediate aftermath of the onset of extinction. Though this echinoid fauna occurs stratigraphically above the main extinction horizon, the occurrence of echinoids likely straddles the PTB, occurring in both the latest Permian and earliest Triassic (Fig. 2). The Tesero Member at the Tesero Section is characterized by basal oolitic beds, followed by an interval of oolitic and bioclastic grainstones and packstones interbedded with marly lenses, mudstones and microbialites (Posenato, 2009). The stratigraphic placement of the echinoid bearing samples is shown in Fig. 2. Two of the samples are from beds interpreted as microbial mats and represent shallow-water settings (CNT10 and CNT11; Noé, 1987; Posenato, 2009). CNT10 contains a fauna dominated by the brachiopod *Orbicoelia* (=?*Crurithyris* of Broglio Loriga et al., 1988), while CNT11 is dominated by *Teserina nerii* (Posenato, 2011). These shallow water brachiopod dominated assemblages in the Tesero section, despite occurring after the onset of the extinction event, yield faunal components characteristic of the Permian. Coeval benthic assemblages from a deeper-water setting (e.g., the Bulla section) are dominated by the bivalve *Eumorphotis,* and show a taxonomic composition similar to those occurring in the Early Triassic, when only a few eurytopic taxa were able to survive in heavily stressed marine conditions (e.g., Posenato, Holmer & Prinoth, 2014; Foster et al., 2017). The assemblages of the echinoid-bearing “mixed fauna” beds at the Tesero section thus record an ephemeral shallow water refuge for marine stenotopic taxa (Broglio Loriga, Neri & Posenato, 1986; Posenato, 2009) (Fig. 2). Echinoid remains are also known from the underlying Changhsingian Bulla Member of the Bellerophon Formation (Broglio Loriga et al., 1988; Posenato, 2009), though their taxonomic affinities are not well characterized.

In the western Dolomites (including the Tesero section), the extinction of stenotopic marine organisms occurred within a stratigraphic interval in the Tesero Member ranging from the contact with the underlying Bulla Member to the lower *H. parvus* Zone. The lower limit corresponds to the basal centimeters of the Tesero Member, which records the onset and peak of the extinction event, represented by a dramatic drop in fossil abundance and the loss of most foraminifers and large brachiopods (Posenato, 2010). The upper limit of the extinction interval is located within the lower *H. parvus* Zone, where the last stenotopic taxa occur rarely (Farabegoli, Perri & Posenato, 2007; Posenato, 2009; Posenato, 2010). The echinoid bearing beds (CNT6-CNT11A) occur stratigraphically above the onset of extinction, indicating that, at least in the Tesero section, echinoids survived the onset of extinction and associated environmental change. The microbialite-rich interval from which echinoids were collected at the Tesero section has been correlated with Beds 27a-b of the Meishan Section in South China (Farabegoli, Perri & Posenato, 2007). The stratigraphically lower echinoid bearing beds at Tesero (CNT6, CNT8, and CNT10) and beds 27a-b at Meishan are within the *H. changxingensis* Zone (Jiang et al., 2007; Lai, Jiang & Wignall, 2018) which is located within the “Survival Interval” at Meishan, where relict Permian taxa survived for ∼60,000 years up to the *I. staeschei* Zone (Shen et al., 2018). This correlation, together with the co-occurrence of echinoids and the “mixed” faunal assemblages at Tesero (Posenato, 2009), seems to indicate that the Tesero echinoids represent holdover taxa from Permian pre-extinction faunas. The delicate nature of the echinoid test recovered (Fig. 3), the occurrence of these echinoids in a microbialite, and the occurrence of articulated brachiopods (Broglio Loriga, Neri & Posenato, 1986), indicates that these taxa were not reworked from older strata and represent an autochthonous assemblage.

### Echinoids and the end-Permian mass extinction

#### Stem group echinoids and disarticulated material

It has been recently demonstrated that stem group echinoids survived the aftermath of the end-Permian extinction event and populated Mesozoic seas until at least the Middle or Late Triassic (Thuy, Hagdorn & Gale, 2017; Thompson et al., 2018). That we find disarticulated spines that display stem group echinoid morphologies (Figs. 6H–6L) in the immediate aftermath of the extinction should thus be no surprise. A current limitation to the understanding of echinoid macroevolutionary dynamics following the end-Permian extinction concerns the general rarity of articulated specimens in the late Permian and Early Triassic. This is exemplified by the fact that there are currently only three species known from relatively well-preserved echinoid tests from Lower Triassic strata (Linck, 1955; Kier, 1968b; Godbold et al., 2017). Despite the rarity of well-preserved specimens, disarticulated echinoid plates and spines are abundant throughout Lower Triassic strata (Broglio Loriga, Masetti & Neri, 1983; Schubert & Bottjer, 1995; Moffat & Bottjer, 1999; Rodland & Bottjer, 2001; Fraiser & Bottjer, 2004; Nützel & Schulbert, 2005; Mata & Woods, 2008; McGowan, Smith & Taylor, 2009; Hofmann et al., 2013; Pietsch et al., 2019; Foster et al., 2018; Foster et al., 2019a; Foster et al., 2019b), though they are typically identified in thin section (e.g., Noé, 1987; Krystyn et al., 2003; Posenato, 2009; Foster et al., 2019a; Foster et al., 2019b). A potential source of “hidden diversity” comes from this disarticulated material (Twitchett & Oji, 2005; Pietsch et al., 2019) as echinoid coronal plates, like the ossicles of other echinoderm groups, display morphological features characteristic of both higher, and lower, taxonomic levels (Gordon & Donovan, 1992; Nebelsick, 1992; Nebelsick, 1995; Nebelsick, 1996; Donovan, 2001; Thompson & Denayer, 2017). Analysis of disarticulated material in Triassic strata has provided novel insight into the diversity and distribution of a number of echinoderm groups during the end-Permian extinction event (Thuy, Hagdorn & Gale, 2017; Reich et al., 2018).

At Tesero, we have shown that disarticulated material has provided novel insight into the pattern of echinoid extinction in the immediate aftermath of the extinction event. The presence of stem group echinoids in beds CNT6 and CNT8 indicates that the stem group echinoids did not go extinct at the initial onset of extinction, which took place about 251.94 Ma (Burgess, Bowring & Shen, 2014) and was responsible in some sections for the extinction of trilobites, rugose corals, and fusulinids (Wang et al., 2014). Instead, stem group echinoids persisted into “mixed fauna” beds, and occur stratigraphically above the initial onset of extinction (Fig. 2). Though stem group echinoids did unequivocally survive the EPME, their abundance and diversity appears to have been severely reduced during the extinction event, and they were likely minor constituents of post-Palaeozoic ecosystems globally (Thompson et al., 2018). At least at Tesero, stem group echinoids appear to have survived the initial onset of the extinction, and may have experienced heightened rates of extinction during potential subsequent pulses of extinction (Song et al., 2013), or during the widespread ecological catastrophes that characterized the rest of the Early Triassic.

#### The duration of Eotiaris through the extinction event

*Eotiaris* is a well-known constituent of late Permian echinoid communities, being known from the Wuchiapingian Zechstein reefs of Germany (Reich, 2007), the UK (Hollingworth & Pettigrew, 1988; Smith & Hollingworth, 1990), and Poland (Peryt et al., 2016). The presence of a species of *Eotiaris* in the Tesero Member is thus indicative of similarities to other Permian echinoid faunas, and further supports the idea that the Tesero echinoids represent a short-lived holdover fauna, which survived the initial environmental change associated with the extinction event (and may have temporarily reappeared in the fossil record in the Siusi Member (late Griesbachian–Dienerian; Broglio Loriga, Masetti & Neri, 1983)). The occurrence of *Eotiaris* during the extinction interval in the Tesero Member also signals similarities to another Tethyan echinoid occurrence known from after the onset of the extinction. This additional occurrence comes from an undescribed species of *Eotiaris* reported from the earliest Griesbachian of South China (Godbold et al., 2017). This species is known from an echinoid concentration of numerous semi-articulated specimens and differs from other latest Permian and Early Triassic echinoid occurrences in that it comes from an upper slope deposit, interpreted to represent an environment below 150 m of water depth. Though the depth of this occurrence differs from other recorded post-extinction occurrences, the palaeoecological distribution of these specimens is similar to the Tesero *Eotiaris* described herein, in that they are also closely associated with microbialites. Species of *Eotiaris* were thus present in the aftermath of the EPME in both shallow settings (e.g., Tesero; Noé, 1987; Posenato, 2009) and in the deeper water environments represented by the Shangsi section (Godbold et al., 2017). Though only these two occurrences of *Eotiaris* are known from beds located above the onset of environmental change and peak extinction, they do signal that the genus was potentially capable of inhabiting a variety of palaeoenvironments, from shallow deposits to deeper water slope deposits. It has been mentioned that echinoderms survived the adverse effects of the EPME in deeper water refugia (Godbold et al., 2017; Thuy, Hagdorn & Gale, 2017). Clearly the occurrence of the echinoids at Tesero indicates that, at least initially, echinoids did survive in shallower water environments as well. This rules out deeper water environments as a sole refuge for echinoderms during the onset of the EMPE. This does not, however, discount deeper water environments as a longer-term refuge, harboring echinoderm taxa throughout the duration of the Early Triassic.

*Eotiaris*, or other miocidarids like it, are thought to represent the ancestors of cidaroid echinoids (Kier, 1968a; Smith & Hollingworth, 1990; Smith, 2007; Kroh & Smith, 2010; Thompson et al., 2015). Cidaroids are abundant and fairly morphologically diverse in the Triassic (Kier, 1968b; Kier, 1977; Kier, 1984; Smith, 1994), and are still extant today (Mortensen, 1928). If the constituent species of the Miocidaridae were capable of inhabiting numerous different palaeoenvironments, their survival and duration through the EPME may represent an example of macroevolutionary processes operating at different scales (Jablonski, 2007). In this case, the ability of constituent miocidarid species to inhabit distinct palaeoenvironments (though this does not necessitate that the constituent species were generalists), may have conferred upon the family a wider environmental range, thus making it less prone to extinction (Jablonski, 2001). A similar trend has been observed with regard to biogeography of mollusks at the K/Pg event (Jablonski, 1986; Jablonski, 2008; Jablonski & Raup, 1995), and environmental breadth and biogeographic distribution of Permian–Triassic gastropods (Erwin, 1989). Interestingly, biogeographic range did not appear to confer a selective advantage upon echinoid genera during the K-Pg extinction (Smith & Jeffery, 1998), and breadth of palaeoenvironmental distribution was not a factor conferring survivorship upon K/Pg bivalves (Jablonski & Raup, 1995). If the environmental distribution of species of *Eotiaris*, or other miocidarids, was such that that the genus or family was capable of inhabiting a wide array of palaeoenvironmental settings, this may explain the duration and survival of the Miocidaridae through the end-Permian extinction and environmental perturbation that characterized the Early Triassic.

## Conclusions

The echinoid fauna from the Tesero Member, including the new species, *E. teseroensis*, informs the spatial and temporal setting of echinoid survival through the end-Permian mass extinction interval. Furthermore, it provides clues as to the traits that may have conferred survival upon the miocidarid echinoids, including a potentially wide palaeoecological distribution globally. In the Tesero section and elsewhere during the Permian and Triassic, the miocidarid echinoids also appear to be associated with microbialites.

As with all aspects of echinoid macroevolution during the Permian–Triassic interval, the ability to test and formulate hypotheses is hindered by the rarity of specimens. Identification and analysis of disarticulated material precisely tied into stratigraphic sections, as was the case with the material studied herein, provides a promising new research avenue for untangling the survival and extinction of echinoids during the EPME. Future work collecting and identifying further disarticulated material from uppermost Permian and Lower Triassic strata will likely provide a more sound understanding of the diversity and distribution of echinoids following the end-Permian extinction event.

## Additional Information and Declarations

### Data Availability

The following information was supplied regarding data availability: Specimen measurements are in Table 1. All fossil material is deposited in the “Museo di Paleontologia e Preistoria Piero Leonardi” of Ferrara University (MPL). Specimen numbers are: MPL 8651-1, MPL 8653-2, 8657-7, MPL 8658-6, MPL 8655-2, MPL 8658-3, MPL 8654-1, MPL 8657-8, MPL 8658-1, MPL 8651-2, MPL 8658-8, MPL 8657-1, MPL 8657-2, MPL 8654-3, MPL 8658-4, MPL 8653-4, MPL 8658-2, and MPL 8658-7.

### New Species Registration

The following information was supplied regarding the registration of a newly described species: Publication LSID: urn:lsid:zoobank.org:pub:5B9E9AE3-B43E-44D0-99BE -F92C979CF325 Eotiaris teseroensis LSID: urn:lsid:zoobank.org:act:C14A9C9C-90FB-4F41-9A7B-AF5D0C43A326.
